# Advancing understanding, diagnosis, and therapies for cutaneous lupus erythematosus within the broader context of systemic lupus erythematosus

**DOI:** 10.12688/f1000research.17787.1

**Published:** 2019-03-25

**Authors:** Kristen L. Chen, Rebecca L. Krain, Victoria P. Werth

**Affiliations:** 1 Department of Dermatology, Corporal Michael J. Crescenz VAMC, 3900 Woodland Avenue, Philadelphia, PA 19104, USA; 2 Department of Dermatology, Perelman Center for Advanced Medicine, University of Pennsylvania, 3400 Civic Center Boulevard, Suite 1-330A, Philadelphia, PA 19104, USA

**Keywords:** cutaneous lupus erythematosus, systemic lupus erythematosus, clinical trials

## Abstract

Cutaneous lupus erythematosus (CLE) is an autoimmune disease that can be associated with systemic lupus erythematosus (SLE) symptoms. The pathogenesis of both CLE and SLE is multifactorial, involving genetic susceptibility, environmental factors, and innate and adaptive immune responses. Despite the efficacy of current medications, many patients remain refractory, highlighting the necessity for new treatment options. Unfortunately, owing to challenges related in part to trial design and disease heterogeneity, only one new biologic in the last 50 years has been approved by the US Food and Drug Administration for the treatment of SLE. Thus, although SLE and CLE have a similar pathogenesis, patients with CLE who do not meet criteria for SLE cannot benefit from this advancement. This article discusses the recent trials and emphasizes the need to include patients with single-organ lupus, such as CLE, in SLE trials.

## Introduction

Lupus erythematosus (LE) is an autoimmune disease associated with a broad range of cutaneous LE (CLE) and systemic LE (SLE) symptoms ^1^. In the US and Europe, the incidence of CLE approximates that of SLE, ranging from 2.0 to 7.6 cases per 100,000 persons per year ^2– 5^. CLE is divided into three primary subsets: acute CLE, subacute CLE (SCLE), and chronic CLE ^6^. Chronic CLE is subsequently categorized as discoid LE (DLE), hypertrophic LE, LE profundus, chilblain CLE, and lupus tumidus ^6^. As suggested by these varied subtypes in CLE alone, lupus is a heterogeneous disease, making diagnosis and treatment challenging in some cases. Patients may be recalcitrant to first- and second-line therapies, underscoring the necessity for new treatments. This review will briefly touch on developments in CLE diagnostic criteria, pathogenesis, current treatment options, and challenges faced in drug trials. We will discuss findings in the most recent therapeutic trials for SLE and highlight the need to include patients with isolated cutaneous symptoms who do not meet criteria for SLE.

## Diagnostic criteria

Whereas SLE criteria have been defined, debated, and revised, the development of CLE criteria is just beginning. The American College of Rheumatology ^7^ and the Systemic Lupus International Collaborating Clinics ^8^ have developed two different classification criteria for SLE. However, both exclude many patients with single-organ lupus (that is, CLE) who have moderate to severe manifestations ^9^. In 2013, the 3rd International Meeting on Cutaneous Lupus Erythematosus began a process to achieve consensus on uniform definitions, diagnostic criteria, and classification of CLE ^10^. Having agreed upon the Delphi consensus method, in which a series of iterative questionnaires are anonymously completed by selected experts, international experts analyzed a “pre-Delphi” questionnaire ^11^. They reported a need for a new CLE definition to improve communication of prognostic information and delineate study populations for both observational and interventional trials ^11^. Most recently, the Delphi method was used to begin developing criteria for diagnosing DLE as part of a larger effort to define CLE ^12^.

## Pathogenesis

The pathogenesis of CLE remains incompletely understood but is multifactorial, involving genetic polymorphisms, susceptibility loci, environmental factors such as ultraviolet (UV) exposure and smoking, and the induction of innate and adaptive immune responses ^13^. T lymphocytes are the predominant cells in CLE; however, plasmacytoid and myeloid dendritic cells (pDCs and mDCs, respectively) play an essential role in disease pathogenesis ^14^. Type 1 interferons (IFNs), which are produced largely by dendritic cells and keratinocytes, are critical to the development of CLE lesions and are produced in response to UV light, nuclear antigens, and immune complexes ^15– 17^. They initiate a cycle of cutaneous inflammation by recruiting leukocytes to the skin via inflammatory cytokines, chemokines, and adhesion molecules ^15^. An IFN signature is present in SLE and the CLE subtypes SCLE and DLE, suggesting a shared pathogenesis ^18^.

## Current treatment options

There are several current treatment options for CLE, and antimalarials (that is, hydroxychloroquine, chloroquine, and quinacrine) are considered first-line therapy. About 75% of patients with CLE respond to antimalarial therapy or topical glucocorticoids or both ^19, 20^. Antimalarials act via immunomodulating effects by influencing antigen presentation, stabilizing lysosomes, inhibiting Toll-like receptor signaling, and reducing IFN production by pDCs ^21, 22^. In particular, quinacrine suppresses the Toll receptor–mediated production of tumor necrosis factor-α likely produced by mDC populations ^14, 23^. Owing to the variation in cutaneous response, antimalarials are frequently used in combination for refractory CLE ^24^. However, should patients remain resistant to antimalarial therapy, immunosuppressives (that is, methotrexate, mycophenolate mofetil, and azathioprine) may be used. Mycophenolate mofetil and mycophenolate sodium have been shown to be highly effective and well tolerated in cases of antimalarial-resistant CLE ^25, 26^. When methotrexate was compared with chloroquine in the treatment of cutaneous manifestations of SLE, low-dose methotrexate was determined to be as effective as chloroquine and to have an acceptable toxicity profile ^27^. Thalidomide is another therapy used in antimalarial-refractory CLE. It is an anti-inflammatory agent and immunomodulator that targets cereblon, reducing the zinc finger transcription factors Aiolos and Ikaros and consequently modulating T-cell function ^28^. It has been shown to be efficacious in treating refractory cutaneous interface manifestations of LE ^29^. However, owing to the high risk of polyneuropathy and teratogenicity, thalidomide should be reserved for cases of severely refractory CLE and used at low doses and as short-term therapy ^29– 31^. More recently, lenalidomide, a thalidomide analog, has gained traction as a useful therapy in patients who remain recalcitrant to antimalarials or thalidomide ^32, 33^. It has been shown to be efficacious and safe and importantly does not cause as much peripheral neuropathy ^33, 34^. Despite this, caution should be taken in women of childbearing age as to date there is no evidence demonstrating safety in human fetal development. In summary, the main current therapies for CLE include antimalarials, glucocorticoids, immunosuppressives, thalidomide, and lenalidomide, which typically provide symptom relief.

## Challenges in trial design

Although current treatment for CLE has been effective, some patients remain refractory to treatment or require less toxic therapies or do both. There is a need for safe and effective therapies for these refractory patients. Despite this, no medications have been approved for CLE in over 50 years; this is largely due to problems associated with lupus trial design ^35^. In a recent proposal for optimizing lupus clinical trials, Merrill *et al.* highlighted challenges to developing new treatments ^9^. These include the heterogeneity of lupus itself, the influence of a wide variety of background therapies, the scarcity of patients meeting stringent enrollment criteria, and the limited number of properly equipped trial sites ^9^. However, the development of the Cutaneous Lupus Erythematosus Disease Area and Severity Index (CLASI) has made it easier to evaluate treatment for CLE, and within the past few years several clinical studies and trials using this tool have shown promising results ^25, 36– 46^. Recently, an international group of dermatologists unanimously agreed that the CLASI be used in clinical trials as a measure of skin activity ^47^. Still, with regard to the progress of new therapeutics, only anifrolumab and baricitinib have recently entered into phase III, and many of the remainder failed to meet critical endpoints.

## Recent drug trials

### Belimumab

Belimumab is a monoclonal antibody directed against B-lymphocyte stimulator (BLyS), an immunomodulatory cytokine that stimulates B-cell differentiation and survival ^48^. A multicenter, randomized, controlled, phase III trial assessed the safety and efficacy of this medication, comparing belimumab plus standard therapy with placebo plus standard therapy in patients with SLE ^49^. In this study, belimumab decreased the number of flares and hindered damage progression in patients with SLE ^40, 49^. Belimumab was also found to improve cutaneous disease, such as rash, mucosal ulcers, and alopecia, and patients with musculoskeletal and skin manifestations responded best to this medication ^40, 50^. Despite this, belimumab is US Food and Drug Administration (FDA)-approved solely for the treatment of SLE, as clinical trials did not formally study the effects of the drug on cutaneous disease ^51^. As a result, patients with CLE struggle to access this medication despite its possible efficacy and tolerability for a subset of patients.

### Sifalimumab and anifrolumab

Sifalimumab, an anti-IFN-α monoclonal antibody, was assessed in SLE patients with moderate to severe disease in a phase IIb, randomized, double-blind, placebo-controlled study. The percentage of patients with improvements in CLASI was greater for all sifalimumab doses compared with the placebo, although *Herpes zoster* infections were more frequent with sifalimumab treatment ^41, 48^. Despite such promising results, this trial was discontinued to further assess anifrolumab, an anti-IFN-α receptor monoclonal antibody that binds the type I IFN-α/β/ω receptor (IFNAR), preventing signaling by all type I IFNs ^42^. In a phase IIb, randomized, double-blind, placebo-controlled study of anifrolumab, as in the study of sifalimumab, a greater percentage of patients showed significant improvement in their cutaneous activity as compared with the placebo group, and more of an effect was seen in patients with higher baseline IFN levels ^42, 48^. Although *Herpes zoster* infections were reported in 5.1% and 9.5% of the patients receiving 300 mg and 1,000 mg of anifrolumab, respectively, the most common adverse events included headache, upper respiratory infection, nasopharyngitis, and urinary tract infection ^42^. The success of this clinical trial led to two phase III studies, and the results have yet to be published. Thus far, findings from both sifalimumab and anifrolumab phase II trials have collectively demonstrated a role of many type I IFNs in patients with SLE.

### Baricitinib

In a double-blind, multicenter, randomized, placebo-controlled phase II study, baricitinib, a Janus kinase 1 (JAK1) and JAK2 inhibitor, was assessed ^43^. JAKs are tyrosine kinases that mediate the signaling of several pro-inflammatory cytokines, most of which have been found to be associated with the pathogenesis of SLE ^43^. Although a large number of patients presented with merely mild cutaneous disease, the larger dose of baricitinib (4 mg) significantly improved the signs and symptoms of SLE, especially arthritis ^43^. As expected, serious adverse events were more common with the 2 mg and 4 mg doses of baricitinib than the placebo (10%, 10%, and 5%, respectively); however, no deaths, malignancies, or major adverse cardiovascular events were noted, making baricitinib a rather safe and tolerable oral medication ^43^. As baricitinib is a potential medication for patients with cutaneous disease, further studies are needed to better understand its possible effect on skin activity; it is recommended patients be enrolled when they present with moderate to severe skin disease so that changes in skin activity can be better noted.

### Emerging therapies

Several recent and upcoming clinical trials have been successful in showing an improvement in cutaneous disease in patients with SLE. BIIB059, an anti-BDCA2 monoclonal antibody, is being studied. The antibody, when bound, leads to internalization of BDCA2, a pDC-specific receptor, and inhibits the production of type I IFNs and other inflammatory mediators ^44^. This phase Ib randomized, double-blind, placebo-controlled, multicenter clinical trial has not only confirmed the role of pDCs in SLE but has also shown a decrease in cutaneous disease activity in these patients as compared with placebo ^44^. Because most adverse events are mild to moderate in severity, the results of this study have led to further development of BIIB059.

Another therapy, CC-220, is also being studied. Similar to thalidomide and its analogs, CC-220 is a high-affinity ligand for cereblon with immunomodulatory properties; its administration decreases Ikaros and Aiolos, two transcription factors encoded by their respective susceptibility loci, IKZF1 and IKZF3, which are associated with SLE ^45^. This randomized, double-blind, placebo-controlled, phase IIa dose escalation study showed a strong correlation between improvement in CLASI score and pDC reduction ^45^. Like BIIB059, CC-220 is being developed further in ongoing studies of SLE patients with skin involvement.

Finally, a recent study assessing the safety and tolerability of ustekinumab, an IL-12/23 monoclonal antibody, was performed, as this pathway has also been associated with the pathogenesis of SLE ^46^. Among the patients with at least mild cutaneous disease in this phase II, placebo-controlled study, a statistically significantly greater percentage of patients saw improvement in skin activity with ustekinumab as compared with placebo ^46^. Such clinical trials not only advance our knowledge and treatment of CLE but more importantly have the potential to improve these patients’ quality of life.

## Special considerations for skin drug trials

As these exciting therapies move forward for SLE, we want to underscore the importance of including patients with CLE in these trials and acknowledge special considerations for skin drug trials. Active CLE is associated with a significant impact on quality of life ^52, 53^ and therefore is worthy of therapy. Furthermore, skin disease in patients with SLE is associated with greater accrual of damage, including chronic seizures and scarring alopecia ^54^. Given that the pathogenesis of SLE and CLE is very similar, treatments for SLE should benefit patients with CLE. However, at present, because patients with CLE are often excluded from these trials, efficacious and safe medications, like belimumab, are not FDA-approved for CLE. Thus, patients are not able to easily access medications that have the potential to alleviate the distress and suffering caused by their disease.

When considering including patients with CLE, we acknowledge certain challenges associated with skin clinical trials. First, among patients with cutaneous disease who are enrolled in clinical trials to evaluate CLE, those with moderate to severe skin activity should be included. For instance, in the baricitinib trial, it was difficult to show an improvement in skin disease with the medication as most patients presented with relatively mild disease ^43^. Second, the use of background therapies has led to high placebo response rates. In order to decrease the chances of this occurring, it is recommended that patients with lower placebo response rates, particularly patients with refractory DLE or SCLE, be enrolled in clinical trials, as these patients do not respond well to background medications ^35^. Notably, however, patients with isolated cutaneous disease generally require fewer background medications compared with patients with multi-organ involvement who may be systemically ill. Given these challenges, recommendations previously put forth include smaller, shorter trials and paring down background therapies when appropriate as well as including more discriminatory endpoints ^9^. We support these suggestions with the hope that patients affected by CLE may obtain better therapies.

## Abbreviations

CLASI, Cutaneous Lupus Erythematosus Disease Area and Severity Index; CLE, cutaneous lupus erythematosus; DLE, discoid lupus erythematosus; FDA, US Food and Drug Administration; IFN, interferon; JAK, Janus kinase; LE, lupus erythematosus; mDC, myeloid dendritic cell; pDC, plasmacytoid dendritic cell; SCLE, subacute cutaneous lupus erythematosus; SLE, systemic lupus erythematosus; UV, ultraviolet
